# Wireless Integrated Biosensors for Point-of-Care Diagnostic Applications

**DOI:** 10.3390/s150203236

**Published:** 2015-02-02

**Authors:** Ebrahim Ghafar-Zadeh

**Affiliations:** Department of Electrical Engineering and Computer Sciences, Lassonde School of Engineering, York University, Toronto, ON M3J1P3, Canada; E-Mail: egz@cse.yorku.ca; Tel.: +1-416-736-2100 (ext. 44646)

**Keywords:** CMOS, point-of-care (POC), wireless biosensors (WBS), integrated electrochemical sensors

## Abstract

Recent advances in integrated biosensors, wireless communication and power harvesting techniques are enticing researchers into spawning a new breed of point-of-care (POC) diagnostic devices that have attracted significant interest from industry. Among these, it is the ones equipped with wireless capabilities that drew our attention in this review paper. Indeed, wireless POC devices offer a great advantage, that of the possibility of exerting continuous monitoring of biologically relevant parameters, metabolites and other bio-molecules, relevant to the management of various morbid diseases such as diabetes, brain cancer, ischemia, and Alzheimer’s. In this review paper, we examine three major categories of miniaturized integrated devices, namely; the implantable Wireless Bio-Sensors (WBSs), the wearable WBSs and the handheld WBSs. In practice, despite the aforesaid progress made in developing wireless platforms, early detection of health imbalances remains a grand challenge from both the technological and the medical points of view. This paper addresses such challenges and reports the state-of-the-art in this interdisciplinary field.

## 1. Introduction

The seminal technological breakthroughs achieved in the field of biomedical instrumentation and related technologies, remain powerless in the face on the morbidity of numerous diseases. Yearly, billions of dollars are spent on the management of such diseases. For example, the Food and Drug Administration (FDA) refrains on approving novel antibiotics for fear of breeding new strains of bacteria by fortifying the ones already in hand. As a counter-measure, the National Institute of Health (NIH) rang the Red-Alert as a warning for eventual infectious diseases outbreaks, given the fact that antibiotics are no longer an option [1]. Consequently, the NIH has encouraged research projects aiming at developing bioengineering means to manage such situations. For instance in this direction, nosocomial infections were intensively addressed and the deployment of networks of wireless bacteria sensors in hospitals is being investigated and represents the quintessence of the bioengineering field [2]. Early detection of health threatening factors is a powerful prevention tool that applies to the medical field in its entirety. Hence, the long term goal behind bioengineering is to develop novel micro- and nano-devices for biological and medical purposes in order to develop detection tools for an arbitrary disease and in an ideal case, the very same tools will convey treatment modalities. In this perspective, the long sought for Complementary Metal Oxide Semiconductor (CMOS)-based DNA sequencing chip, has been recently completed through integration of millions of Ion Selective Field Effect (ISFET) transistors with a microfluidic device, and is being commercialized by Ion Torrent^™^ (Life Technologies, New York, NY, USA). The latter device has proven to be paramount for genetic research [3]. Likewise the short-term goal of bioengineering is then to develop new low-cost and user-friendly devices for continuous POC management of diseases. These devices can be divided into three different classes; wearable, implantable and handheld ones. Hitherto and as described in the next section, major leaps have been made by industry in the three categories to different degrees; some of them have undergone successful commercialization while others are still in developmental stages.

Various technologies have been investigated as to their production yield and scalability when implementing mass-production of POC devices. Among those, CMOS offers a distinct low-cost and a very-large-scale-of-integration (VLSI) for control circuits of built-in and monolithically integrated sensors and actuators. Hence, CMOS, besides serving as a support of many applications in communications, media and computation, today emerges as the best candidate to be adopted in bringing POC technologies to a large consumer-market. Thus, far from being an exhaustive report on the POC field in its entirety, this document describes the recent progress made exclusively in biosensors that lend themselves to be implemented in a miniaturized wireless system to finally make integrated WBSs. As integrated wireless technologies have been developed successfully in recent years, they are not the main focus of this paper. Section 2 provides a an overall view of the state-of-the-art in the field, while Section 3 addresses CMOS/fluid related interfacing concerns, followed by a summary in Section 4.

## 2. State-of-the Art in the Field of WBSs

In this section, we review the recent advances achieved in implantable and wearable biosensors conceived for POC applications.

### 2.1. Implantable Biomedical Devices for POC Diagnostics

**Figure 1.**
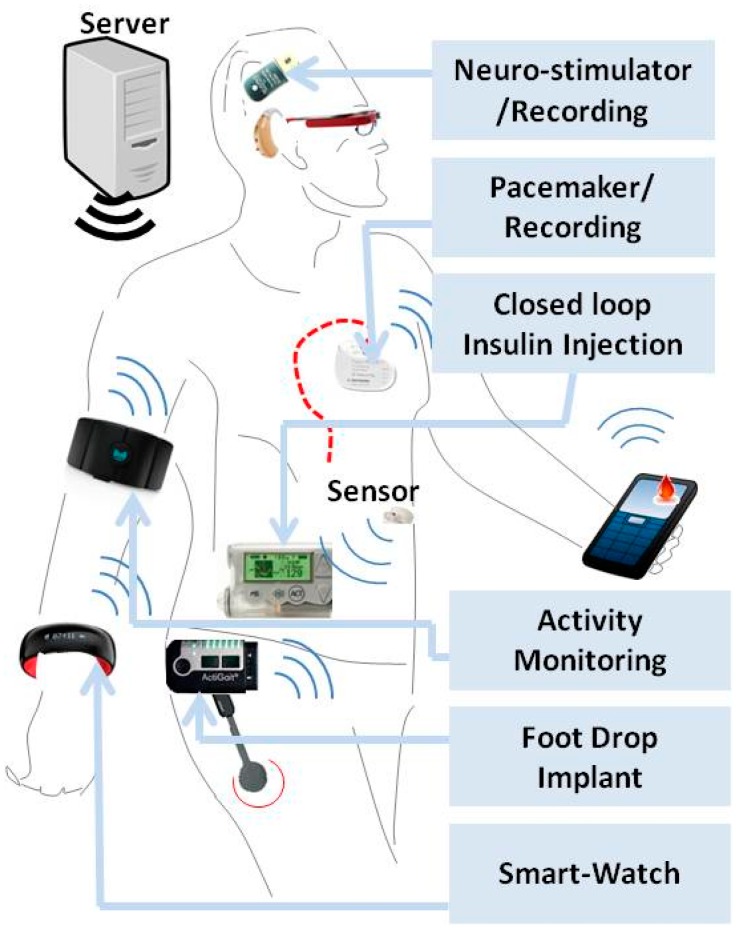
WBS technology for POC diagnostics of various diseases such as epilepsy, diabetes, foot drop using various implantable, wearable and handheld devices. These devices can be controlled by a base server through a wireless system.

**Figure 2.**
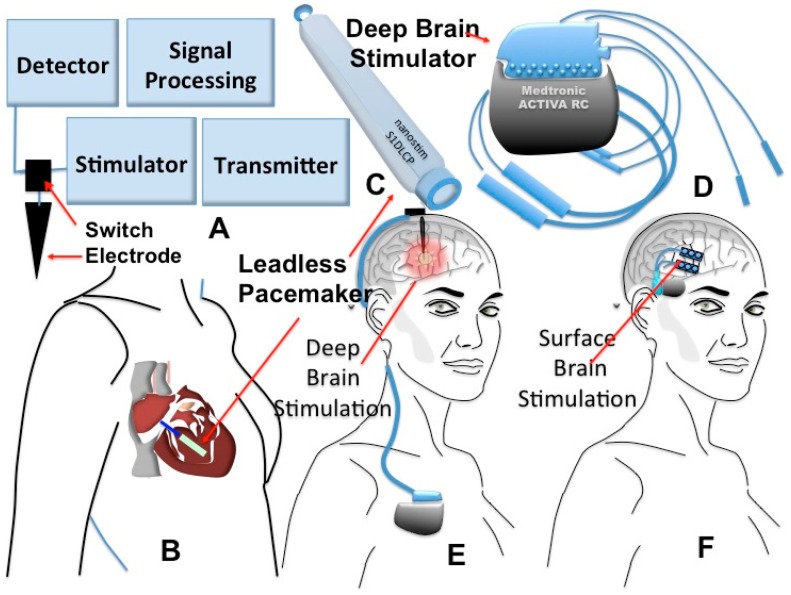
State of the art implantable devices for brain POC applications: (**A**) simplified diagram of implantable system; (**B**) the schematic of new wireless pacemaker placed in the heart; (**C**) the photo of this leadless pacemaker commercialized by Nanosim Inc.; (**D**) brain stimulator commercialized by Medtronics for epilepsy point-of-care purposes implanted; (**E**) under the skull for surface brain stimulation or (**F**) the stimulator placed above the chest for deep brain stimulation.

The field of implanted medical devices is turning into one of the most profitable businesses in USA, as these devices possess great advantages over their classical counterparts, since they offer better improvements to the quality of life of patients with chronic diseases, condemned to permanent and daily use of bio-analytical and invasive tests. Hitherto, many implantable devices have been approved by the FDA for a variety of applications ranging from monitoring of the glycemic index to the detection of heart and brain electrical imbalances and palliation of hearing malfunctions [4–6]. In the beginning, the main role of implanted devices was to replace a missing biological structure through use of prosthesis such as artificial hearts or support damaged anatomical structures like arteries through the use of stents. Hence, as a natural to continuation in this field, miniaturized implantable biosensors for point-of-care diagnostics of diseases come about as the new generation of implantable medical devices that feature higher measurement sensitivities of physiological parameters (Figure 1). As shown in Figure 1, WBSs can be classified in three groups including, wearable, handheld and implantable devices suitable for the detection of various diseases. Among these, depicted in Figure 2A–E, are the commercially available devices for management of brain and heart diseases.

In these devices, as seen in Figure 1, analysis of the recorded signals can for instance, predict the onset of an epileptic seizure and accordingly stimulate the brain from the internal surface of the skull or directly in the deep brain. The recorded signal can be transmitted wirelessly as shown in Figure 2E,F [7,8]. A similar technique is also being employed in novel heart pacemakers. Indeed, it was recently announced that a new pacemaker technology that uses electrical stimulation allows for making miniaturized devices that could be implanted in a minimally-invasive way and most importantly, the wireless device senses heart electrical activity and stimulates it without resorting to the use of leads, as shown in Figure 2B,C ([9,10]). As, to the recording of brain electrical activity, the latter is sensed and the resulting electrical signal could be used for brain stimulation purposes for different diseases such as epilepsy and Alzheimer’s. The main technical challenge in such bio-sensing devices is the type and number of electrodes. Ideally, the ability to access a number of cells around the brain individually can open several research avenues in the study of diseases that affect the Central Nervous System (CNS). However technically, this is not possible due to the damage inflicted to cells by electrodes. Therefore, the main tendency in device manufacturing, when using the current technology, strives to improve the conductivity at the electrode-tissue interface, using for instance, novel materials such as carbon nanotubes or nanowires [7,8]. Also, the functionalization of said electrodes for selectively detecting other factors such as pH or glucose is another sought and important goal.

**Figure 3.**
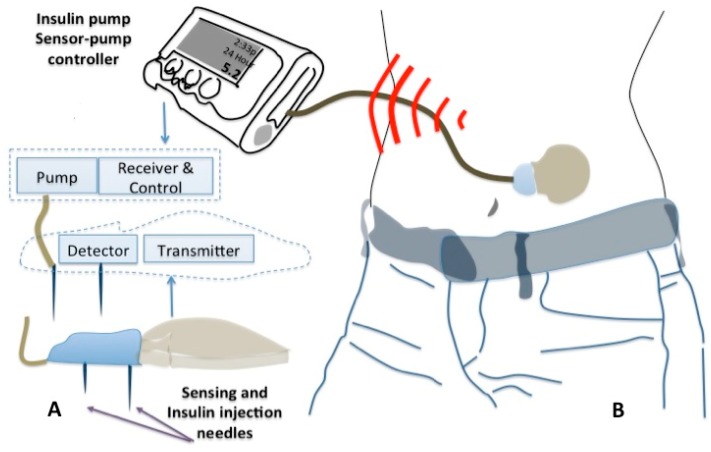
State of the art Implantable devices for diabetes POC applications, (**A**) a simplified diagram of implantable glucose monitoring and insulin injection and (**B**) a schematic of closed loop POC system.

In this direction, novel implantable miniaturized and functionalized electrodes for continuous glucose monitoring have recently received FDA approval for commercialization [11–13]. The system has wireless capabilities and could transmit instructions to a mini-pump and trigger release of regulated and physiologically relevant doses of insulin as shown in Figure 3.

### 2.2. Recent Advances in Wearable WBSs Technologies

**Figure 4.**
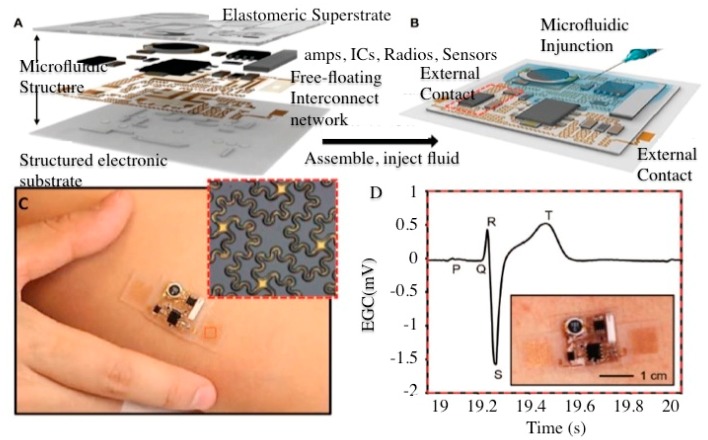
Novel soft elastomeric wireless wearable sensing system (**A**–**B**) schematics of structured elastomeric substrate with free-floating network; (**C**) photo of patched adhesive wearable system and (**D**) the ECG curve recorded with the wearable sensing system (Reproduced with permission from Science [17]).

As described in the last section, modern medical devices have been designed and developed based on micro-sensors that exhibit wireless functionalities taken in the broadest sense, namely data transfer, energy transfer and leadless stimulation [14]. The three aspects allow for real-time continuous monitoring of clinically relevant parameters, battery-less operating mode for long-term use and preservation of the integrity of the sensed/stimulated tissues, respectively. Despite the great capabilities of microelectronic technologies, the packaging of wearable WBS systems attached to skin surfaces remains a challenge given the fact that in such configurations, the WBS/skin interface features cumbersome time-varying dynamics that are extremely hard to counteract [15,16]. To address this inconvenience, among the techniques that resort to standard silicon technology, an article published in *Science* reported the integration of microelectronic devices on a soft elastomeric substrate using microfluidic techniques [17] (Figure 4).

As illustrated in Figure 4A,B, this solution consists of two elastomeric substrates, electronic components and a free-floating strip-line network assembled into a single package. The resulting ultra-thin and wireless adhesive device (Figure 4C,D) has the ability to monitor heart rates and other vital parameters and transmit data in real-time to a cell phone or a computer.

**Figure 5.**
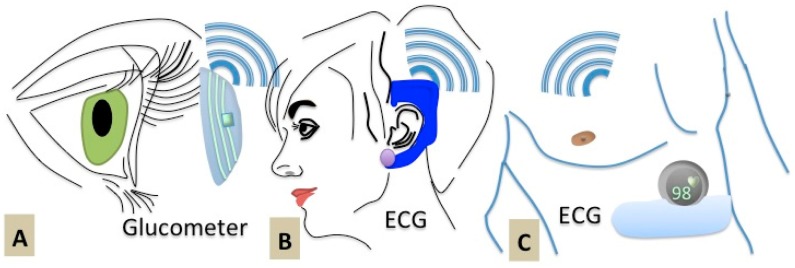
State of art wearable POC devices: schematic of (**A**) contact-lens glucose sensor commercialized by Google and ECG sensors patched on (**B**) chest and (**C**) ear.

Similar efforts have been made by the R&D group of a startup company called Vital-Connect Inc. (Campbell, CA, USA), to encapsulate eight different health-sensing devices within one, single-lead-ECG device, in order to monitor heart rate, heart rate variability, respiratory rate, skin temperature and posture [18] The device is being commercialized in form of a patch, recently approved by the FDA. The sensors can be used for clinical-grade measurements and wirelessly transmitting the information to computers or cell phones. In such WBS devices, the challenges of combining recognition elements and electronic devices still persist. To date, several efforts have been made at developing patch-like biosensors, to detect biomarkers in sweat fluids [19,20] using various techniques, the most promising one being the use of biomarkers that can also be found in the tears fluid (for instance for glucose sensing), saliva and phlegm. The recently publicized active eye contact lens by Google aims at contently measuring the glucose levels in tear fluid (Figure 5A). The successful integration of such devices with RF electronics on one chip could be regarded as the next essential step towards the development of complete and fully functional WBS. Instead, the current market of biosensors is rather promoting the use of electrical sensors that are attached to the chest or the ears in order to exert continuous monitoring of heartbeats and breathing rates (Figure 5B,C) [21,22]. The sensor/skin interface does not bring in any kind of inappropriateness in these anatomical locations.

**Figure 6.**
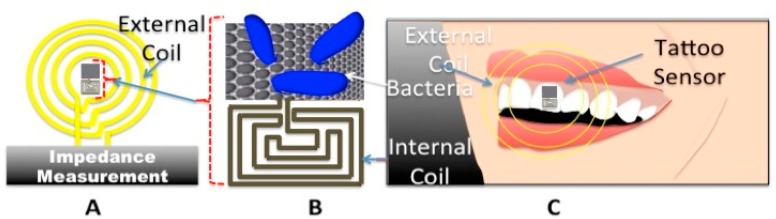
Remote Bio-sensing technique: Simplified diagram of (**A**) sensing technique measuring the impedance change due to the presence of bacteria; (**B**) sensing electrodes on soft substrate; (**C**) attached electrodes on tooth.

On the contrary, development of novel wearable WBS, for quantification of biomarkers remains the main challenge to be taken on by device manufacturers before bringing any viable solution to the market of POC devices. Among very few research works carried out in this context, Mannoor *et al.* developed a graphene-based electrode for a peptide based biosensing platform. The electrodes were functionalized with peptides and used for monitoring of respiration and bacteria detection in saliva [23]. As seen in Figure 6A, the presence of bacteria can be detected in a contact-free mode through a change in resonance of signals are subsequently transferred to the base-station using a RF-link. The device offers a great advantage in real-time and continuous bacteria monitoring on teeth (Figure 6B,C). This passive wireless telemetry system consists of a planar meander-line inductor and an interdigitated capacitive electrodes integrated onto the graphene/silk film and are subsequently transferred onto the surface of a human molar. As described in [23], the proposed graphene sensor functionalized with 38 amino acid sequences, in the order of HSSYWYAFNNKT-GGG-GLLRASSVWGRKYYVDLAGCAKA (GBP–OHP) and GBP in presence of deionized water (DI), erythrocytes and Gram-positive *Staphylococcus aureus*. Such remote sensing techniques can be appended to sophisticated diagnostics methods such as magnetic resonance relaxometry for the detection of doxorubicin-induced cardiotoxicity [24]. Given that the main goal of this paper is far from being the covering of the entire set of WBS devices, we will exclusively cover and discuss sensing techniques that have been implemented in standard microelectronic technologies.

From this perspective, some appealing works have been reported on the subject. To name a few, an implantable WBS for the real-time *in-vivo* measurement of pH fluctuations has been developed [25]. This technique resorts to an oxidized single-walled carbon nanotubes used as ox-SWNTs functionalized with a conductive polymer called poly-aminoanthracene or PAA used as a sensor. A wirelessly powered and passive Radio-Frequency Identification (RFID) tag was used to transmit pH data through simulated skin. The functionality of the proposed device was shown by changing the pH values. This device offers the advantage of making a viable means for early detection and potential prevention of bacteria colonization of surgical implants. Detection in this case is accomplished through monitoring of the biofilm-induced acidosis.

### 2.3. Handheld Bio-Sensing Systems

**Figure 7.**
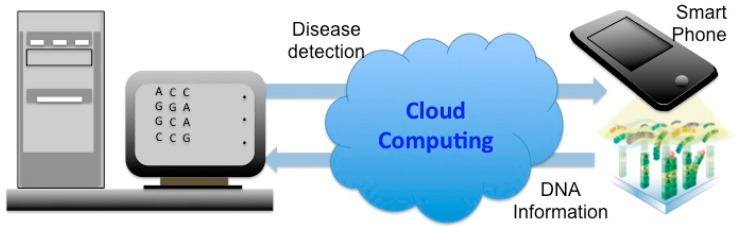
State of art technology of wireless handheld POC technology: Avatar DNA, for automatic identification using an optical machine-readable DNA icon on microarray. The telepathic DNA-DNA hybrids inscribed (on-chips) and identified by camera of smartphone with application software.

Handheld biosensors are one of the most sought for analytical components to be added to the POC arsenal. Their field of applications ranges from classical glucometers to advanced devices for magnetic immunoassay tests [26,27]. A recently reported technique uses magnetic nanoparticles and immunoassay protocols to measure concentrations of target molecules [28]. A disposable biosensor cartridge is inserted into this hand-held analyzer for the measurement and the data are transmitted wirelessly to the base-station. Another handheld system is the Stat-Strip Glucose Hospital Meter System that represents the first FDA-approved glucose meter that was for use in Intensive Care Units (ICUs) [29]. As these handled systems include a display screen and a Random-Access-Memory (RAM) and can be connected to computers using wired data-links, wireless communication techniques do not play an essential role in most of these devices. In fact the wireless techniques are advantageous when information should be sent directly to the medical center to start decision making processes regarding an emergency or when continuous monitoring of the patients critical state is needed. To make a case for the usefulness of wireless data transfer one should consider the genetic sequencing that is emerging as the ideal method for disease diagnostics [30,31]. To date tremendous effort has been geared toward sequencing of DNA, DNA analysis and gene detection. For instance, Oxford Nanopore Inc. (Oxford, UK), by proposing the first handheld DNA sequencing system has revolutionized biotechnology [32]. The genetic information extracted from cells should be recombined on supercomputers for gene and subsequently disease detection purposes. For this reason, wireless techniques were the best suited for operating information transfer and can be integrated with the miniaturized DNA sequencing device. Among the few successful attempts in this direction, Park *et al.* developed a novel DNA sequencing hybrid system [33]. Figure 7 illustrates how the digital DNA icons inscribed on a chip of a microarray can be automatically captured with a mobile phone of a camera and information can be directly accessed by software. This technology employs fabricated DNA image codes for digital and automatic identification and data capture (AIDC) to rapidly transmit information to remote databases. In conclusion of this section, one could assert that the use of the current CMOS-based DNA sequencing technologies, will soon be spurring great improvements in the market of WBS-based POC devices. An application of this wireless technology is the disease detection using genetic codes.

As a summary of this section, it is noteworthy that the development of fully integrated WBS devices using standard microelectronic technology such as CMOS, for implantable and/or wireless applications is in an incipient and immature developmental stage. The current state-of-the-art in this field described in the last section endorsed by the literature leads us to conclude that wireless sensing, data transfer and energy transfer techniques have successfully been applied in the field of POC diagnostics and the main challenge facing future developments is the difficulty of achieving full integration of sensors and circuits along with their associated recognition elements. Now, among various optical, magnetic and electrochemical sensing techniques, emphasis is put in this paper on the devices that employ electrochemical methods, for they are easily amenable to fully integrated and miniaturized POC devices that feature low-cost and ease of scalability in their production.

## 3. Toward Fully Integrated CMOS Based Biosensors

Standard microfabrication technologies are the best candidate for the development of integrated POC systems. The foundries offer efficient mass-production platforms that lower the cost of microelectronics, making the price low enough to make electronic products affordable to the end-users. As to microchip scale of the integration, today’s foundry processes have reached the threshold of 15 nm (minimum feature of Field-Effect-Transistors—FETs) and hence they allow for the making of highly dense systems featuring millions of active elements, which as a whole, form an integrated circuit (IC) used nowadays in a variety of industrial applications. In current uses, the advantage of standard microelectronic technologies, particularly CMOS, is the fact that they allow for creating monolithic integration of large numbers of micro-scale sensors/actuators along with their electronic circuitry. Such an approach allows the whole device to become capable of incarnating sensing techniques such as those based on optical [34,35], magnetic [36] and electrochemical including impedometric [37], capacitive [38] and ISFET [3] techniques. Among these techniques, only electrochemical sensors offer the advantages of label-free detection, suitable for WBS purposes. For this reason, the focus of this section is placed on electrochemical biosensors as the best candidates for wireless integrated bio-sensing applications.

### 3.1. Electrochemical Biosensors

**Figure 8.**
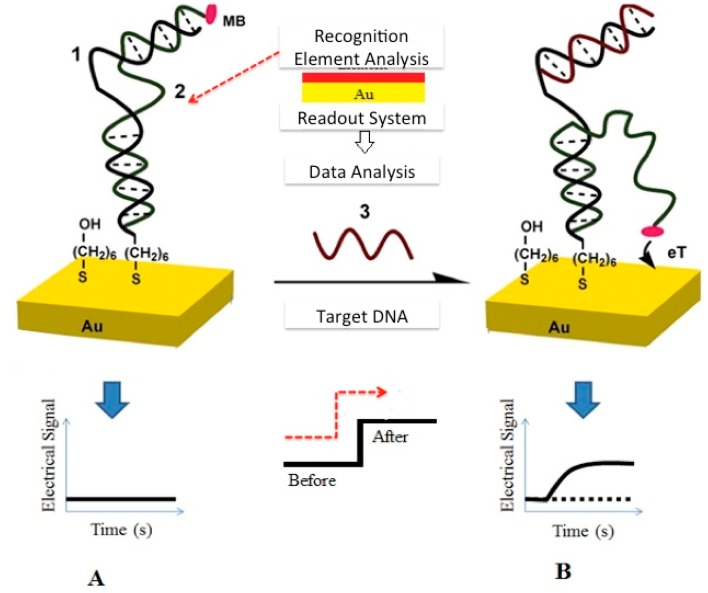
Electrochemical sensor: (**A**–**B**) schematic of an electrochemical E-DNA sensor consisting of recognition element, sensing electrode and interface circuit (**A**) prior and (**B**) after detection of target sample (Reproduced with Copyright permission from (2006) National Academy of Sciences, USA [38]).

An electrochemical sensor (ES) relies on electrical charge transfer between an electrode and the target biological or chemical sample. Typically, an electrochemical sensor consists of a set of sensing electrodes and an interface readout circuitry (Figure 8). The interface circuit is designed to detect and transduce minute electrical signals transduced by the electrodes. The electrodes are functionalized with biological receptors also called recognition elements (REs) capable of selectively binding to targeted molecule or cell surface-receptors. The main role of REs in electrochemical sensing is to undergo biophysical changes that occur as a result of their interactions with target molecules present in the analytes. These changes are then transduced to fluctuations in electrical parameters like electrical charge, dielectric property or conductivity. Figure 8 shows an example of a newly developed ES, based on a specific mechanism called target-induced strand-displacement that results in the production of a relatively small output current [39]. This method is mainly used for DNA detection with potential uses in handheld WBS.

### 3.2. Sensing Electrodes

The set of electrodes in an ES is meant to be put in contact with the targets carried by sample fluids and they typically, consist of three kinds of electrodes—a working-electrode (W-E), a counter-electrode (C-E) and a reference-electrode (R-E). This configuration yields two signals and ground reference as opposed to the intuitive classical setting where only two electrodes are used. This three-electrode measuring scheme appears to be useful in avoiding the polarization problems found in the ground electrode of a dual-electrode setup. Another example of a recent electrochemical sensor is a contact lens features three integrated electrodes for the detection of biomarkers including glucose in tears [40]. A tiny CMOS chip is employed to detect the impedance changes between the electrodes and wirelessly transmits the sensing data using a micro-coil integrated in the same contact lens.

### 3.3. Electrochemical Techniques

**Figure 9.**
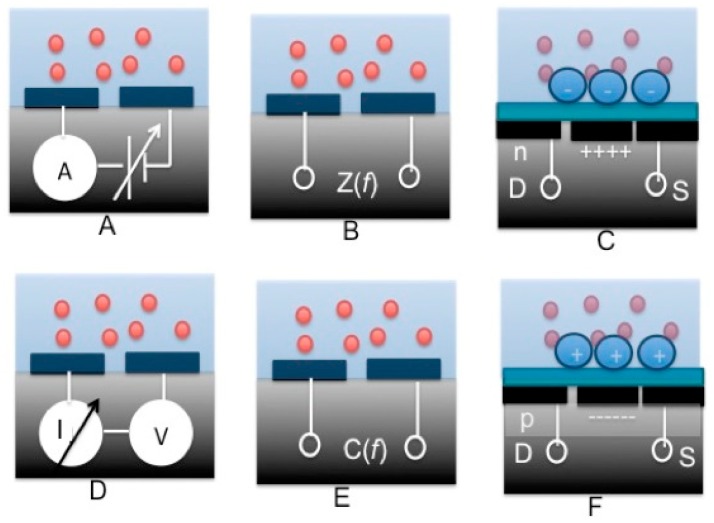
Interfacing techniques for electrochemical sensors: (**A**) voltometric; (**B**) impedometric; (**C**) p-channel ISFET; (**D**) amperometric; (**E**) capacitive; and (**F**) n-channel ISFET.

Electrochemical sensors can be classified into three categories; voltammetric (amperometric), potentiometric and impedometric (including capacitive and conductometric) techniques as shown in Figure 9A–F.

Voltametry (or amperometry) is a powerful method used for conducting quantitative determination of biological materials that draw in close to the electrodes. In this technique, the transduced current is measured continuously while the relative difference in the electrical potential of the electrodes is being swept in time in a linear manner. Figure 9A,D illustrates the voltammetric and the amperometric methods used in ESs, respectively. On the contrary, as depicted in Figure 9D, an amperometric technique is similar to voltammetry, except that it is neither the electrical potential between the electrodes that is measured while the magnitude of the electrical current is being swept in time. Furthermore, the readout system of an ES should be capable of performing frequency dependent measurements of the relative changes in impedance, capacitance or conductance between the electrodes (Figure 9B,E). The impedance of a thin layer that forms above the electrodes is modulated by the physiochemical properties of the biological and chemical materials present in analytes. The variations in these physiochemical properties can also be measured through potentiometric techniques. Potentiometry is a readout technique suitable for use in sensing applications where the cumulative electrical charge created by free electrons and ions (positive or negative) present in the scrutinized solution, creates a difference in the electrical potential on top of a dielectric layer. The potential across the latter is then modulated and on the other side of the dielectric material, the induced difference of electrical potential is measured with no current flowing in the sample fluid. An Ion-Selective Field Effect Transistor (ISFET) is a good example of that kind of sensors that successfully incarnate potentiometric techniques. As they represent highly sensitive devices, ISFETs can measure changes in the electrical potential of fluid solutions while the transistor’s gate current is null. Figure 9E and 9F shows two types of ISFETs with n- and p-channels, respectively. As described therein, the minute changes of electrical properties around the insulated gate could be detected with dedicated readout circuitries. It goes without saying that further electronic stages (analog circuits) should be placed downstream to collect and amplify the minute analog signals, followed by other stages (digital circuits) that convert the amplified output to digital signals. Additionally, the digital stages are also required to develop multiplexers, primary on-chip signal processing and wireless transmission units.

### 3.4. CMOS-Based Bio-Sensing Structure

**Figure 10.**
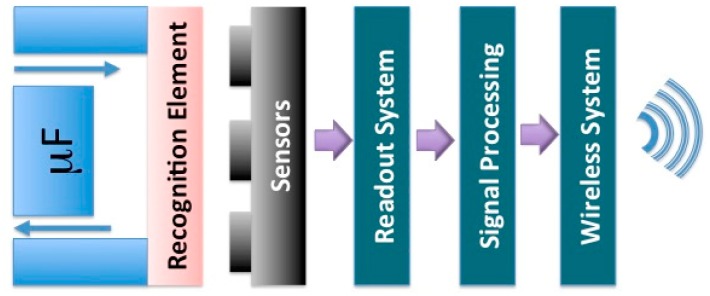
A simplified model of integrated biosensors including an interface circuit connected to front end devices (microelectrodes). These electrodes are coated with RE in order to transduce the biological activities to physical changes. The main building blocks are used in wearable, handheld and implantable biosensors, however the microfluidic (μF) structure is used in handheld POC systems.

Standard CMOS technology allows for a myriad of configurations including embedded sensors and/or actuators. Integrated circuitry and ease of programmability and control of the sensors promote CMOS foundry process to the technology of choice for monolithic integration of sensing devices (e.g., microelectrodes) along with their readout systems. In fact, a holistic system-based approach to CMOS electrochemical sensor design integrates front-end devices (e.g., ISFET), interface circuits and biological and chemical recognition elements seamlessly in a straightforward way (Figure 10) [41–43].

**Figure 11.**
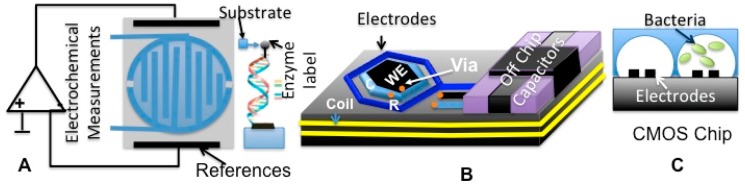
A CMOS biosensor: DNA detection, amperometric detection of (**A**) DNA hybridization using CMOS interdigitated microelectrodes functionalized with DNA probes; (**B**) a schematic of an implantable glucose monitoring, capacitive bacteria growth monitoring; (**C**) schematic of a differential method bacteria growth monitoring.

The electrode can be realized through standard CMOS processes, however for some applications, further post-fabrication micromachining procedures should be applied accordingly. For instance, gold (Au) electrodes are required most of the time for their known chemistry and functionalization procedures that are applied to bind different recognition elements (RE) on said electrodes [44,45]. Given the fact that chemical vapor deposition (CVD) of (Au) is not supported by CMOS, other post-processing steps of CMOS-based sensors need to be applied in order to make an ES. In many cases, a microfluidic structure could also be integrated with CMOS chips to direct the fluids toward the ES sites while it prevents direct contact between solutions and the remaining part of the readout circuitry. Among the variety of biological applications of CMOS-based ES, one could consider DNA analysis, metabolic monitoring, metal-ion sensing, glucose monitoring, bacteria growth monitoring and tracking of the proliferation of cancer cells [46,47]. Electrochemical DNA sensors constitute efficient and low cost devices for genetic screening and detection. As shown in Figure 11A,B, a conventional DNA sensor operation is based on the chemical interaction (hybridization) between DNA molecular targets in solution and a layer of known DNA fragments attached to an electrode in order to produce electrical signals. The attachment of known DNA strands on electrodes requires a certain linker that plays the role of an interface between surface and target recognition molecules [48]. The hybridization between known and unknown DNA strands could be detected using electrochemical-sensing techniques.

### 3.5. CMOS Circuits and Systems Design for Biological Applications

As hinted above, a CMOS chip could be designed to detect the presence and hybridization between known and unknown DNA molecules as shown in Figure 11A [49]. This figure illustrates the development of an electrochemical sensing device on CMOS chips that can be used as handheld DNA detection devices. Another application for CMOS–based ES is in the measurement of glycemic index, relevant to the management of diabetes. In this perspective, microelectrode surfaces are typically functionalized (chemical treatment steps leading to the binding of a RE) using glucose oxidase enzyme (Gox) or other types of enzymes. Recently, an implantable miniaturized glucose sensor has been developed on CMOS chips to perform continuous *in-vivo* monitoring of the glycemic index. Such a continuous glucose monitoring system features wireless transmission capabilities and consists of CMOS- integrated RF transponder, readout interfacing system and sensing electrodes (Figure 11B) [44].

In another field of applications, settings for monitoring of cellular behaviors such as growth and proliferation are shown to be practically amenable to ESs-based impedometric, capacitive and conductive techniques. These uses of ESs paved the way for unprecedented monitoring capabilities where networks of WBSs are needed, like in agriculture for monitoring of crops infections, environmental monitoring (e.g., bacteria detection in hospital buildings) and continuous monitoring of disease metastasis (e.g., cancer cell detection). Figures 11C shows an on-chip bacteria growth monitoring technique using a CMOS chip fabricated in a 0.18 μm process. Here, CMOS-based ESs could be employed for a variety of applications using a specific RE, however, since the focus of this review is geared towards the engineering aspects of electrochemical sensors, a full expansion on the subject of the biological and the chemical protocols proper to the field of surface functionalization with REs, could be found in the related references. In the remaining paragraphs of this section, the electrochemical voltammetric and impedometric methods are described in Sections 3.5.1 and 3.5.2 respectively. Among potentiometric methods, particular attention will be paid to ISFET techniques in Section 3.5.3.

#### 3.5.1. CMOS-Based Voltametric Sensors (Amperometry)

In voltametric techniques, by applying a DC voltage to an electrode, the relationship between current and voltage becomes a function of the chemical/biological properties of species present in the sample. In this simple sensing method, a feedback circuit is used to apply a desired potential across the working electrode (WE) and the circuit interface used to measures the resulting current. The integration of the required circuitry for voltametric measurements on the very same chip that harbors the microelectrodes, significantly decreases the need for using current and voltage references and above all, the effect of parasitic capacitance that arises from excessive usage of interconnections (electrodes and the measurement system) on the readout path, becomes drastically insignificant. Hence, voltametric or amperometric circuitries can successfully and most efficiently be integrated in CMOS chips along with low-noise-amplifiers (LNAs) circuits to eliminate the non-idealities caused in principle by the flow of stray currents in sample solution. Table 1 shows different configurations that could be developed in a CMOS process and applied to various biological and chemical applications including DNA detection. Further, a chronocoulombmetric DNA detection is performed based on the oxidation and reduction of labeling molecules attached to hybridized DNA target strands [48]. Some known drawbacks to this method are; the background and offset signals originating from the presence of the electrochemical agents exacerbated by the double layer capacitance that forms between electrodes and electrolytes. As a follow up to the work reported in [48], a novel DNA micro array developed in CMOS 0.5 μm technology was reported to successfully overcome the aforesaid drawbacks.

In order to eradicate these non-idealities, the main innovation in this novel design is the use of a differential measuring scheme, a fast integration algorithm and an optimized value in the triggering voltage-steps [49].

In another approach, a redox-cycling-based electrochemical array of 128 sensors is presented in [50]. By applying oxidation and reduction potential on two interdigitated electrodes, the current flows through both electrodes. This current has two main components; the current initially generated by the enzyme label and the current of the redox-cycling. Besides, and due to electrochemical artifacts, an offset current may also contribute to the total signal. Hence, instead of measuring the absolute value of currents, it is its first derivative with respect to time that yields useful information. The total measuring time is of the order of seconds to minutes. Using the same detection principle employed in [50], an improved chip of an array of DNA sensors was realized with as many analog to digital convertors as the number of sensors [51]. Here, again, in order to eliminate the offset current in the signals picked up by the proposed high dynamic range-sensing chip, it is the first derivative of the current with respect to the measuring time that is of interest. In the same work, a very simple integrated current-to-frequency converter is utilized. The accuracy of the conversion circuit is mainly determined by the process variations of the capacitors and also the comparator used in the analogue-to-digital converter (ADC). Also, another redox-cycling-based sensing configuration is presented in [52] in which, target molecules are conjugated with ferrocene radix labels. It is worth mentioning here, that the most common redox labels used in DNA detection are ferrocene, K_3_Fe[(CN)]_6_^3−/4−^, Ru (bpy)_3_^3+/2+^, Os (bpy)_3_^3+/2+^ and Methylene Blue. The presented work has two main features: first the operation doesn’t require any washing steps, so the measurement could be performed repeatedly and in real-time. Second, it provides a quantitative measurement using a potentiometric measuring scheme. The surface density of hybridized DNA pairs is be determined by integrating the area under the curve of the ferrocene (Fc) redox, generated current. Additionally, a CMOS-based DNA sensor based on a cyclic voltametry technique was presented in [53]. Here, the ferrocene labels were used as redox species since they undergo reaction-induced changes over a well-defined range of electrical potentials. The advantage of this method over intercalation-based approaches (reversible inclusion of a molecule between two other molecules) is that in the latter methods the amount of probe-target hybridization on the surface of the WE cannot be measured. However, in the proposed cyclic voltage method, the quantitative detection of DNA hybridization is carried out by integrating the area enclosed by the curve of the redox current and subtracting the contribution of the background. More of a reason, in support to the idea of resorting to CMOS processes in bio-sensing, the main advantage of CMOS technology for electrochemical sensing in particular, is the fact that it offers a low-power, compact area and integrated low-noise circuitries necessary for conducting amperometric sensing experiments. However, when an amperometric sensor is sensitive to noise, the WE can be connected to a ground potential, and that makes the sensor less sensitive to noise and interference parasitic contributions [54]. Furthermore, since only a small number of passive elements are used in [54] for measuring the currents, the circuit has potentially lower level of noise. The proposed device is then suitable for implantable and portable applications. In addition to the described methods, there is a number of other proposed architectures summarized in Table 1 [55–58].

#### 3.5.2. CMOS Impedometric Sensors (Capacitive and Conductive)

The embedded sensing electrodes realized in the topmost metal layer of CMOS chips, in addition to voltammetry (and amperometry). They can be employed for measuring impedance, capacitance or conductivity of the targeted biological sample. In fact, an impedometric sensor requires the same sensing electrode configuration like the one used in voltametry, but a different interface circuitry is needed. As shown in Table 2, hitherto, many research papers have reported the advantage of CMOS technology for the development of portable, high precision and low cost impedometric, capacitive, and conductive biological sensors, however less attention has been paid to the measurement of the pure conductivity of biological samples. This is due to the significant effect of the double layer capacitance that forms on the surface of the electrodes.

##### Impedometric Techniques

**Figure 12.**
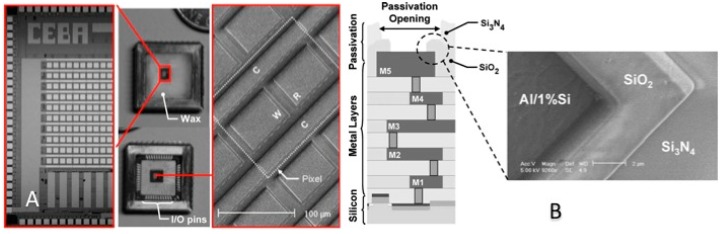
(**A**) CMOS based ES consisting of an array of amperometric sensors with three W, R and C electrodes; (**B**) multilayers CMOS process to create Al electrodes (Reproduced with permission from IEEE, [58]).

Among the efforts put in view of implementing on-chip measurement schemes of impedance, Hassibi *et al.* demonstrated the design, implementation and functionality of an impedometric sensor in the 0.18 μm CMOS process using a 1% Al to Si metallic layer for making the WE, CE and RE structures, as shown in Figure 12 [58]. It should be stressed that Al is not as widely used as Au in biochemistry and an oxidation layer under normal environmental conditions easily covers it.

However, considering that this work focuses on integrated circuit design, the specification of the proposed ES array makes it suitable for many biochemical detection methods, including those used to detect DNA and proteins. In this way, Levine *et al.* reported the making of another integrated impedometric ES array implemented through a 0.25 μm CMOS process for DNA detection [59]. Further post-processing techniques like etching and sputtering procedures were performed to implement Au-based electrodes instead of the standard aluminum electrodes. DNA detection can also be performed using capacitive sensing techniques.

##### Capacitive Techniques

Depending on the biological or physiochemical properties of the sensing electrodes, the impedance change of occurs due to the variation of capacitive or resistive properties. Therefore, a low complexity capacitive or conductive readout technique can be employed instead of impedometric technique. For instance, the hybridization of DNA molecule was shown to be detected by a capacitive sensor. Based on the technique proposed by Stagni *et al*., the unknown capacitance change due to DNA hybridization is charged and then discharged when the applied pulse with a certain voltage amplitude and time period becomes positive or negative, respectively [60]. The precision of the proposed time-based capacitive sensor depends on several factors including the charge concentration of electrolytes and formation of the double layer capacitance above the said electrodes. In fact, the measured capacitance always demonstrates a combination of double layer capacitance and changes in dielectric properties of the sample. Capacitive sensors have efficiently been employed in other fields of application, including chemical solvent and bacteria growth monitoring. Ghafar-Zadeh *et al.* developed and successfully implemented a precise readout scheme having charge-based capacitance measurement (CBCM) at its core. The method proved efficient in the measurement of femto-Faraday (fF) capacitance changes between the sensing electrodes exposed to biochemical samples [61].

**Figure 13.**
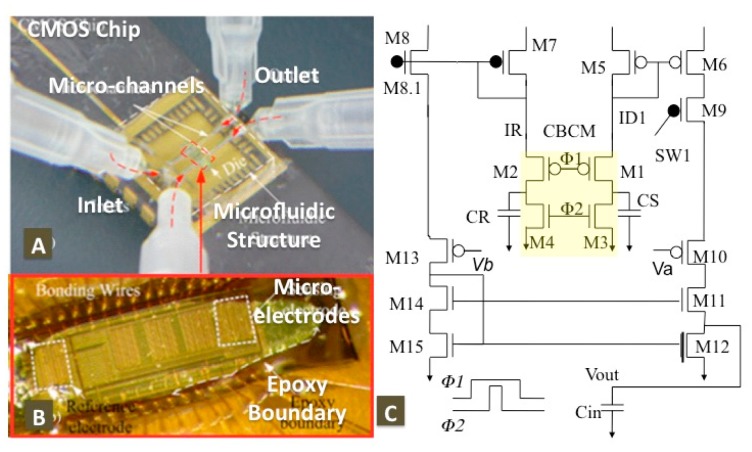
Core-CBCM Capacitive Sensor: photograph of (**A**) Die including (**B**) an array of three sensing capacitances and Integrated microfluidic CMOS system and (**C**) Core-CBCM Capacitive interface circuit (Reproduced with permission from IEEE [46]).

**Figure 14.**
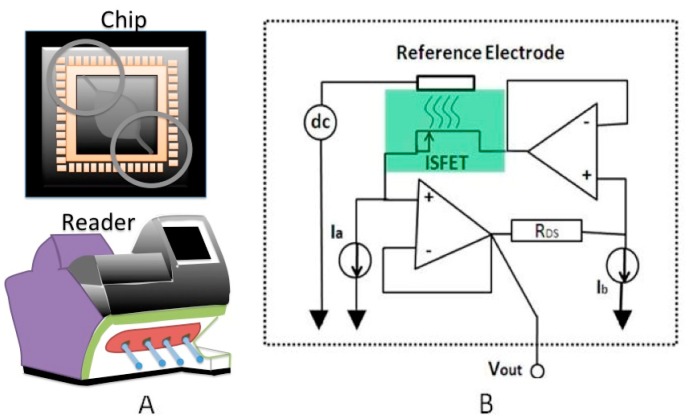
State of art CMOS ISFET technology: (**A**) illustration of a sample preparation and a readout system for DNA sequencing; (**B**) Simple ISFET interface circuit.

A core-CBCM interface circuit consists of the CBCM structure (M1–M4), current mirror amplifier (M5–M8), unity gain current differentiator (M10–M15) and a multiplexing switch (M9). The proposed core-CBCM capacitive sensor has been developed in 0.18 μm CMOS process and integrated with microfluidic structure as shown in Figure 13A. In the aforementioned work, CBCM was utilized with much more simplicity compared to other capacitive interface circuitries in extracting sensing-driven capacitance changes prior and post-injection of analytes. Furthermore, the capacitive sensor (Figure 14C) can be used to monitor bacterial growth using integrated microfluidic CMOS system (Figure 14A) and interdigitated microelectrodes (Figure 13B). Figure 13B also shows the epoxy encapsulation of die everywhere except the sensing electrodes. Therefore, CMOS-based capacitive sensors are key devices useful in a numerous biological and chemical applications such as virus detection and tracking cancer cell proliferation [45].

##### Conductometric Techniques

Conductometric circuits are used in analysis of biochemical reactions in which, determination of the dissociation of various acids is put under scrutiny [62]. In these techniques, the resistance (or conductance) of the sample solution between two electrodes is measured as a function of a specific biological or biochemical activity [63,64]. Additionally, Yao *et al.* reported the use of a CMOS chip for conductometry-based detection of bacteria using a conductance to frequency conversion scheme [62]. Precisely, the electrical resistance of the test sample is converted into a frequency-modulated pulse. Subsequently, the group demonstrated successful detection of very low initial concentrations of *E. coli* bacteria (4 × 10^2^ colony forming units (CFU/mL)) with and without its specific receptor T4 bacteriophage.

#### 3.5.3. CMOS-ISFET Sensors

Ion-Selective-FET (ISFET) can be described as a conventional Metal Oxide Semiconductor Field Effect Transistor (MOSFET) device with a floating gate. Here, a reference electrode inserted in an aqueous solution is electrically connected to the device through an ionic conductive electrolyte [65,66]. A thin-membrane isolates the semiconductor active regions from electrolytes, so as any gradients in the concentration the ionic charge, modulates the ISFET threshold voltage (V_T_). It is worth mentioning that the post-processing of fabricated standard chips can increase the cost of devices dramatically. Fortunately, ISFETs are fabricated in a standard CMOS process without necessitating any post-processing step, a fact that reduces their cost and allows the downsizing of these devices to more compacted form compared to their impedometric or conductive counterparts [67,68]. As depicted in Figure 14A, the metal layers in a CMOS chip are employed to construct via between the CMOS passivation layers and the poly-silicon semiconductor. Furthermore, CMOS processes support the formation of epitaxial thin silicon nitride and/or silicon oxide for use as insulation layer(s) since these two materials are widely used for sensing applications due to their linear responses. In fact, ISFETs could be employed for various applications such as DNA detection, extracellular imaging and cellular activities (Table 3).

As listed in Table 3, in addition to DNA analysis, ISFETs are proven to be ideal platforms for the detection of neuronal activities [3,71], for cellular population monitoring [71] and for environmental studies such as water safety monitoring [72]. Moreover, silicon nitride is often used in CMOS-based ISFET devices for many of these applications as endorsed by the references in Table 3. The insulation layers are sometimes covered with other thin films such as gold or titanium oxide for the creation of layers of recognition elements above the CMOS sensor [73,74]. Insulation layers could be coated with recognition element (RE) such as DNA strands. Further, REs are used to detect the presence of molecules such as the negatively charged DNA strands selectively. It is worthwhile to mention those CMOS-based ISFET sensors are the first and the only commercialized WSB devices (Ion Torrent Inc., New York, NY, USA) for electrochemical sensing applications [75,76]. As seen in Figure 14A, this device features more than 20 million ISFETs integrated with the same number of microfluidic chambers, is used to carry out the sequencing of the full genome after the sample preparation. In each of said chambers, each time an arbitrary nucleotide is binded to a DNA strand by a polymerase, a hydrogen ion (H^+^) is released as a consequence. This reaction results in a pH increase that can be detected by the ISFET. The sample preparation is an important process for DNA sequencing. This process consists of DNA extraction, micro-bead integration into DNA sequencing and DNA amplification or so called polymerase chain reaction (PCR). Figure 14A show a fully automated system for sample preparation and detection using a hybrid microfluidic/CMOS chip used as a disposable array of sensors.

##### Interface Circuits

Measurement of ISFET threshold variation is performed through the simple circuit shown in Figure 14B. This circuit consists of a source-drain follower topology with a constant drain current and a constant drain-source voltage [77–80]. In a Field Effect Transistor (FET) operating in the saturation region, the drain current (*I_D_*) is a function of threshold voltage (*V_T_*), drain-source voltage (*V_DS_*) and gate-sources voltage *(V*_GS_) as it is explicated in the following equation:

(1) $$I_{D}=k\frac{W}{L}\cdot {\left(V_{GS}-V_{T}\right)}^{2}\left(1+qV_{DS}\right)$$

where *k* and *q* are the Boltzmann (1.3806488 × 10^−23^ J/K) and the electron charge (1.602176487 × 10^−19^ C) constants. By assuming that *I_D_* and *V_DS_* are constants, *V*_GS_ should compensate for the change in *V_T_*. A circuit topology can be developed with the adjusted *I_D_* and *V_DS_* to constant well determined values using the current sources I_a_ and I_b_. A constant gate potential *V_G_* can be provided by dropping a DC constant voltage on the reference electrode. Therefore, *V_T_* = −*V_S_* and consequently *V_out_* = *V_T_*. Furthermore, a fully differential ISFET interfacing circuit that incorporates two simple readout circuits could significantly improve the results by eliminating the similar non-idealities inherited from the circuit electronics. The output voltages are differentiated using an OPAMP [81–84]. Regardless of the significant advantages of using operational amplifiers to create linear relationships between threshold voltages and subsequently the electrolytes’ charges, the use of OPAMPs along with ISFETs in the interface circuits results in high power consumption and crowding of larger silicon areas, especially when the sensing platforms features a large number of ISFETs. An alternative solution is to multiplex a large array of ISFETs using a number of switches accordingly [72].

##### Non-Idealities Associated with ISFETs

The most important non-idealities of CMOS-based ISFETs derive from the presence of trapped charges. These charges can randomly change the values of *V_T_*. A practical solution to remove the excess charge is to apply UV radiation [85]. To date, tremendous effort has been put into developing new readout techniques capable of eliminating the undesirable effects of trapped charges or other sources of noise. For instance, the dominant low frequency (1/f) noise mostly results from Si/SiO_2_ interface. Moreover, an effective method is to use p-MOSFET instead of the commonly used n-channel, for effective noise reduction [86]. Additional roles of the interfacing circuit consist of eliminating the effect of the temperature fluctuation or thermal drifts for it has been argued that temperature variations are considered to be the main environmental factor affecting the performance of an ISFETs. Also, several compensation techniques have been developed to cancel out the effect of temperature variation [87–89]. For instance, temperature compensation has been considered as the main part of ISFET interface circuit design indicated in the first ISFET paper published by Bergveld *et al.* [66].

In this section, the recent advances in electrochemical sensing techniques were reviewed for various applications including point-of-care diagnostics [90,91], biological analysis platforms and water safety [47,70]. Electrochemical sensors are the best candidates as the biological sensing structures to be incorporated with wireless circuitries in order to develop integrated wireless biosensors for life science applications.

## 4. Conclusions

In this paper, the recent progress in the field of point of care diagnostic (POCD) devices were described. The main challenge in developing wireless integrated biosensors is the development of integrated sensors and circuits on the same chip. We also put forward the advanced CMOS techniques used to develop electrochemical sensors. Among these, electrochemical impedometric, amperometric, voltometric and ISFET methods were comprehensively described, with further details of interface circuits and non-idealities, which can be compensated mostly using specific circuitries. The road map of point-of-care diagnostic technologies is the development of RF integrated sensors and actuators functionalized with recognition element for specific sensing purposes and CMOS will play a very critical role to microchip scale integration technology in future development in this field.

## Figures and Tables

**Table 1.** CMOS based Voltammetry/Amperometry techniques.

**Sensor**	**CMOS**	**Linearity**	**Output**	**Electrodes**	**Reference**
Chemical	0.18	500 pA–10 μA	Voltage (CT)	Single	[48]
DNA	0.5	NA	NA	24 × 16	[49]
DNA	0.5	1 pA–0.1 μA	Voltage (CT)	16 × 8	[50]
DNA	0.5	1 pA–0.1 μA	Frequency	16 × 8	[51]
DNA	0.25	N.A	Voltage (CT)	4 × 4	[52]
DNA	0.25	N.A	Voltage (CT)	4 × 4	[53]
Chemical	0.18	1 nA/1 μA	Frequency	Single	[54]
Chemical	0.5	±500 fA	Voltage (DT)	2 × 2	[55]
Chemical	0.5	0.10–8.0 mM	Voltage (CT)	Single	[56]
LoC	0.5	NA	Voltage (CT)	32 × 32	[57]
DNA	0.35	NA	Voltage (DT)	24 × 24	[58]

**Table 2.** CMOS based impedometric or capacitive techniques.

**Application**	**CMOS**	**Technique**	**Facts**	**Reference**
DNA	0.18	Impedometric	Aluminum SE ^1^	[58]
DNA	0.25	Impedometric		[59]
DNA	0.1	Capacitive	TBM ^2^	[60]
Chemical Solvent	0.18	Capacitive	CBCM ^3^	[61]
Bacteria Growth	0.18	Capacitive	Differential CBCM	[46]
Cancer Tracking	0.18	Capacitive	SE	[47]
Bacteria Growth	0.35	Conductometric	SE	[62]

1 SE: Sensing Electrode;

2 TBM: Time based Measurement;

3 CBCM: Charge Based Capacitance Measurement.

**Table 3.** Summary of CMOS ISFET applications.

**Sensor**	**CMOS**	**Membrane**	**Electrodes**	**Reference**
Extracellular imaging	0.35	Si_3_N_4_	16 × 16	[69]
Water microorganism	N.A.	Si_3_N_4_	single	[70]
Cell population	4	Si_3_N_4_	12	[71]
SNP detection	N.A.	Si_3_N_4_	Single	[3]
DNA sequencing	0.35	Tantalum oxide	1.5–13 M	[72]
DNA/protein molecules	0.5	Gold	single	[73]
DNA hybridization	4	Si_3_N_4_	single	[74]
DNA detection	0.35	Si_3_N_4_	40	[75]
